# A Structural Equation Model of HIV-Related Stigma, Racial Discrimination, Housing Insecurity and Wellbeing among African and Caribbean Black Women Living with HIV in Ontario, Canada

**DOI:** 10.1371/journal.pone.0162826

**Published:** 2016-09-26

**Authors:** Carmen H. Logie, Jesse I. R. Jenkinson, Valerie Earnshaw, Wangari Tharao, Mona R. Loutfy

**Affiliations:** 1 Factor-Inwentash Faculty of Social Work, University of Toronto, Toronto, Ontario, Canada; 2 Women’s College Research Institute, Women’s College Hospital, University of Toronto, Toronto, Ontario, Canada; 3 Dalla Lana School of Public Health, Social and Behavioural Health Sciences, University of Toronto, Toronto, Ontario, Canada; 4 Human Development and Family Studies, University of Delaware, Newark, Delaware, United States; 5 Women’s Health in Women’s Hands Community Health Centre, Toronto, Ontario, Canada; Liverpool School of Tropical Medicine, UNITED KINGDOM

## Abstract

African and Caribbean Black women in Canada have new HIV infection rates 7 times higher than their white counterparts. This overrepresentation is situated in structural contexts of inequities that result in social, economic and health disparities among African and Caribbean Black populations. Economic insecurity is a distal driver of HIV vulnerability, reducing access to HIV testing, prevention and care. Less is known about how economic insecurity indicators, such as housing security, continue to influence the lives of women living with HIV following HIV-positive diagnoses. The aim of this study was to test a conceptual model of the pathways linking HIV-related stigma, racial discrimination, housing insecurity, and wellbeing (depression, social support, self-rated health). We implemented a cross-sectional survey with African and Caribbean Black women living with HIV in 5 Ontario cities, and included 157 participants with complete data in the analyses. We conducted structural equation modeling using maximum likelihood estimation to evaluate the hypothesized conceptual model. One-fifth (22.5%; n = 39) of participants reported housing insecurity. As hypothesized, racial discrimination had significant direct effects on: HIV-related stigma, depression and social support, and an indirect effect on self-rated health via HIV-related stigma. HIV-related stigma and housing insecurity had direct effects on depression and social support, and HIV-related stigma had a direct effect on self-rated health. The model fit the data well: χ^2^ (45, n = 154) = 54.28, p = 0.387; CFI = 0.997; TLI = 0.996; RMSEA = 0.016. Findings highlight the need to address housing insecurity and intersecting forms of stigma and discrimination among African and Caribbean Black women living with HIV. Understanding the complex relationships between housing insecurity, HIV-related stigma, racial discrimination, and wellbeing can inform multi-level interventions to reduce stigma and enhance health.

## Introduction

African and Caribbean Black (ACB) women remain overrepresented in Canada’s HIV epidemic with new infection rates 7 times higher than their white counterparts. [1] This is similar to the experience of African American women in the United States, who have HIV infection rates close to 4 times that of white women. [2] Heightened HIV vulnerability among women and ethno-racial minorities, and people at the intersection of these identities, is situated in structural contexts of social, economic and political inequities. [3, 4] Structural factors such as economic insecurity have a complex and indirect association with HIV risk, operating distally to reduce access to HIV testing, prevention, and care. [3, 5–10]

Prior research indicates that ACB women experience higher rates of HIV-related stigma then non-racialized women. [11, 12] Marginalized women experience discrimination in the form of classism, racism and sexism, among others.[13–16] This marginalization may be exacerbated for ACB women living with HIV (WLHW). The convergence of gender and racial inequities produce unique and distinct experiences of HIV stigma among ACB women that reflect sexist and racist stereotypes. [11, 17, 18] Little is known about dimensions of economic insecurity, such as housing insecurity, and its interplay with stigma and health among ACB WLHW. This study aims to address this knowledge gap by modeling the relationships between housing insecurity, racial discrimination, HIV-related stigma, and wellbeing (depression, social support, self-rated health[SRH]) among ACB WLWH. We first review the effects of HIV-related stigma and racial discrimination on people living with HIV (PLHIV), and then explore housing insecurity and wellbeing among PLHIV. We theoretically situate this study in the fundamental causes of health; finally we report the study design and findings.

### HIV Related Stigma, Racial Discrimination, and Wellbeing

Structural perspectives articulate that stigma processes occur across micro, meso and macro levels, and include labeling, loss of status, and discrimination in contexts of unequal power dynamics. [19, 20] Social environments with chronic and acute stressors—such as stigma and discrimination—enhance susceptibility to health problems. [21] There is a rich body of literature highlighting deleterious impacts of HIV-related stigma on mental and physical health, as well as social support, among PLHIV. [22–26] Processes by which HIV-related stigma can effect psychological and health outcomes are complex. For example, *internalized stigma*—acceptance of negative views about PLHIV—may be associated with shame, which in turn contributes to depression. [26, 27] *Enacted stigma*, negative treatment from others, and *perceived stigma*, awareness of negative social norms and attitudes towards PLHIV, contribute to psychological distress and reduced wellbeing. [26] The convergence of internalized, enacted and perceived stigma may reduce health care services utilization and medication adherence, in turn compromising physical wellbeing of PLHIV. [27]

The deleterious impacts of racial discrimination on health are well documented; systematic review findings highlight the impacts of racism on poorer mental health and reduced health behaviors. [28] Racial discrimination refers to inequitable and oppressive systems founded on ethno-racial differences, including beliefs, attitudes, exclusion, harassment, and institutional policies and practices. [29, 30] Few studies, however, have examined racial discrimination and wellbeing among PLHIV. [22] One study in the Canadian context examined gender and ethnicity differences in HIV-related stigma; the gender-ethnicity interaction term was significant and Black women reported among the highest HIV-related stigma scores. [11] Race is also a known determinant of housing insecurity. [31] The impacts of race on socioeconomic status are shaped by reduced access to education and employment opportunities, and, in a large part, residential segregation. In the US numerous studies have demonstrated that race and ethnicity powerfully impact the housing markets that individuals have access to. [31–33] This is largely attributed to discrimination and limited social capital of poor and minority individuals. [31, 32] Health is not simply connected to race indirectly through socioeconomic status, but racism and discrimination also have direct impacts on health status, for example, through neighbourhood segregation which leads to inequitable service provision. [31] In Canada, ethno-racial groups have been identified as high-risk for homelessness, although public discussions of homelessness are often silent on the issue of race and racism as determinants of homelessness. [34]

For ACB women, these factors are heightened if they are also immigrants. Canadian studies show that racialized immigrants are likely to face stigma and discrimination both on account of their race and on their immigration status.[35–38] Immigrants may be stigmatized through ‘othering’, a process that differentiates persons based on perceived differences [39] and contributes to marginalization and social exclusion [40]. Immigrants, in general, experience poorer wellbeing than the general population with elevated risk of depression, chronic pain, and other somatic complaints. [41, 42] Psychotic disorders may be elevated among immigrants post-migration. [43–45] In Canada exposure to racism and discrimination has been shown to have negative impacts on the mental health of immigrants and refugees. [46–48] Racism also presents as a barrier to accessing housing for immigrants. Teixeira (2006) found that recent Angolan and Mozambican immigrants in Toronto, Canada encountered barriers to securing affordable and adequate housing, including prejudice and discrimination based on their race and skin colour. [49] The experiences of immigrant PLHIV may be compounded by limited knowledge of local health systems, poverty, and immigration status. [50–52] A study from the United Kingdom found that stigma and discrimination because of their migrant status greatly impacted the ways in which migrants PLHIV accessed HIV-related care and support. [51] The experiences of immigrant ACB WLWH are understudied in the Canadian context.

### Housing Insecurity and Wellbeing Among People With HIV

Across the globe complex causal pathways contribute to health inequities among people with lower socio-economic status (SES) in comparison with their higher SES counterparts. [53–56] In Canada individuals with lower SES are four times more likely to report fair or poor health than higher-income individuals. [57, 58] Longitudinal studies reveal that Canadians living in the poorest 20% of urban neighborhoods die earlier than other Canadians. [57, 58] In a US population-based study, socio-economic disparities accounted for a significant proportion of health disparities across multiple health outcomes including AIDS mortality. [59]

The link between housing insecurity and HIV is well documented, and complex. Homelessness and HIV interact in two distinct ways: homeless individuals are at a greater risk of HIV infection, women in particular, [60, 61] and homelessness can have negative impacts on the health of PLHIV. [62–64] To manage their chronic condition, PLHIV must follow drug regimens, secure ample nutrition and rest, and access frequent monitoring and social support by health professionals. [65] This is difficult to do in the face of housing insecurity. [66] Housing insecurity is particularly important to examine among PLHIV, as it is associated with faster progression from HIV to AIDS and AIDS mortality. [67] However, more research is needed in the Canadian context of health and social correlates of SES disparities among PLHIV.

Housing insecurity is associated with deleterious physical and mental health outcomes among PLHIV. [66] In a longitudinal US study of PLHIV with unstable housing, Riley et al. [68] found unmet subsistence needs were associated with poor mental and physical health outcomes among HIV-positive men, and negative impacts on mental and gynecological health among WLWH. [69] A 2004 Canadian study found HIV related illnesses to be a leading cause of death among homeless women aged 18–44. [70] A systematic review of the effects of housing status on health-related outcomes among PLHIV found a positive association between housing stability and improved health-related outcomes, including: medication adherence, health and social services utilization, and health status. [65] One study in this review examined mental health status, finding housing stability not to be significantly associated with mental health functioning. [71] Both the author of the study and authors of the review highlight the need for further research that includes psychological health-related outcomes. [65] Furthermore, only 1 of the 29 studies in this review included Canadian populations, underscoring the need for further research on housing insecurity among PLHIV in this context. Qualitative research reported that housing instability, homelessness and transience pose barriers to accessing health services and social support, in particular for ACB WLWH in Toronto, Canada. [72, 73] Another qualitative study highlighted that ACB WLWH experiencing housing insecurity face increased stressors, including barriers to supports. [74]

Social support and social networks often have positive influences on health, buffering the adverse physical and mental health effects of stressors. [75, 76] Persons with robust and high quality social networks have decreased mortality and morbidity. [77] Social support has also been shown have beneficial health effects for PHLIV, including increased quality of life, coping skills, and treatment success. [78] Poverty, however, is associated with reduced social support. Kawachi and Kennedy [79] reported that income inequality reduces the ability to achieve social cohesion. A recent U.S. study demonstrated that among the urban poor, poverty-specific stressors are associated with poor health, and social supports may not have as great a health impact as previously understood. [80] Associations between housing insecurity and social support among PLHIV are less understood. This is a salient area to examine as HIV-related stigma is widely associated with reduced social support among PLHIV. [22]

Housing insecurity has been linked with higher rates of HIV-related stigma. [22, 81] Precariously housed PLHIV may experience intersecting forms of stigmatization based on their HIV positive status and their housing status. Wolitski et al. [62] note that in the US homeless PLHIV may experience higher HIV-related stigma due to stereotypes that they are “responsible” for their HIV infection, as well as due to intersections with stigma already experienced based on race, ethnicity and housing status, among other factors.

Housing insecurity has also been linked with broad experiences of discrimination, not limited to HIV. Persons with lower SES may perceive themselves to experience higher rates of stigma and discrimination, particularly in Western contexts where values of meritocracy can place personal responsibility for poverty on individuals. [82–84] In a US study, poverty was correlated with allostatic load and perceived discrimination. [85] This study used a general, unspecified discrimination measure to assess inequitable treatment based on any aspect of one’s background or identity.

We did not find quantitative studies with PLHIV that examined associations between housing insecurity and forms of stigma beyond HIV-related stigma—such as racism. More research is needed to conceptualize the complex relationships between different forms of stigma and discrimination, housing insecurity, and wellbeing among ACB WLWH, particularly in the Canadian context.

### Theoretical Approach

Our study was guided by fundamental cause theory. Link and Phelan [86] developed fundamental cause theory to conceptualize the linkages between distal factors, such as social contexts and SES, and persistent health disparities. Fundamental causes refer to factors associated with multiple health inequities across varied contexts and manifold risk factors. [86, 87] Another key aspect is that fundamental causes, such as SES, reduce access to resources—such as money, status, social support, housing—that could reduce vulnerability to disease as well as limit its consequences. [86, 87] Hatzenbuehler, Link and Phelan [88] also highlight the role of stigma as a fundamental cause, whereby multiple stigmatized identities (race, gender, sexuality, etc.) can lead to limited access to resources and increased vulnerability to poor health. The relationships between housing insecurity with health outcomes may be further complicated when multiple forms of discrimination are considered.

**Fig 1:**
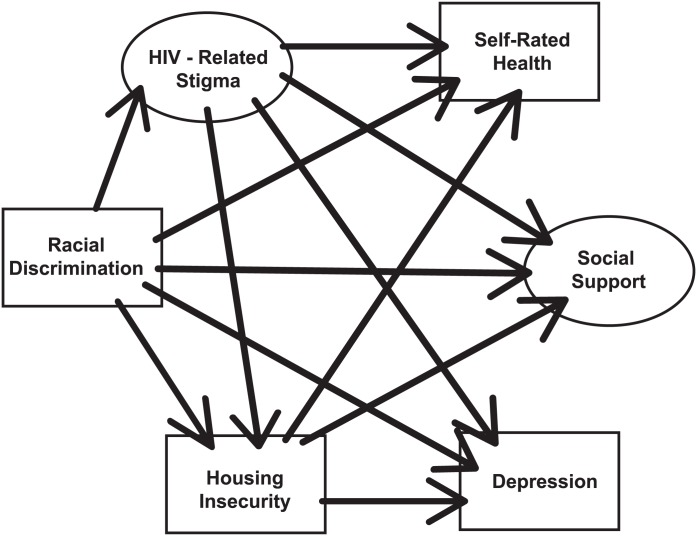
Tested conceptual model of the relationship between HIV-related stigma, racial discrimination, housing insecurity and wellbeing among African and Caribbean women living with HIV in Ontario. Fig 1 depicts hypothesized relationships between variables analyzed in the model. Solid lines represent hypothesized direct effects. Ovals represent latent variables, and rectangles represent observed variables.

The purpose of this study was to extend prior research on stigma and health correlates of housing insecurity among WLWH by examining pathways between a) forms of social inequities (racial discrimination → HIV-related stigma; racial discrimination and HIV-related stigma → housing insecurity), and b) social inequities (HIV-related stigma, racial discrimination, housing insecurity) and indicators of wellbeing (depression, social support, self-rated health). We developed a conceptual model building on prior research, depicted in Fig 1. As illustrated in the model, we hypothesized that a) racial discrimination would increase HIV-related stigma; b) HIV-related stigma and racial discrimination would be associated with increased housing insecurity; c) racial discrimination, HIV-related stigma, and housing insecurity would contribute to reduced wellbeing, specifically higher depression, lower social support and lower self-rated health.

## Methods

We implemented a cross-sectional survey in 5 Ontario cities to explore stigma, health and social support factors associated with food and housing insecurity among ACB WLWH. The survey completion time was approximately 90 minutes, and participants were provided referrals and resources for social and health services in their region upon completion. Participant inclusion criteria were being 18 years or older, self-identifying as a HIV-positive woman, English speaking, and identifying as being an African or Caribbean Black (ACB) woman. Respondents provided written informed consent prior to survey participation and received a small honorarium. Research Ethics Board approval was obtained from Women’s College Research Institute at Women’s College Hospital, University of Toronto.

We worked with nine peer research assistants (PRA) who were ACB WLWH, employed or volunteers with community organizations serving PLHIV in 3 Ontario cities. Following adaptations made from recommendations following a pilot test, the questionnaire was implemented by PRA between June 2010 and January 2011. Participants were recruited using purposive and convenience sampling from community-based ethno-racial and HIV organizations, community health centers, clinics and hospitals. We aimed to recruit 200 participants with an equal proportion of African and Caribbean ethnicity. There is no standardized sample size for SEM; [89] with 6 variables, including 2 latent variables with a combined 9 indicators, this sample size included at least 10 cases per variable.

### Measures

We assessed socio-demographic variables including income, age, education and ethnicity. We assessed housing insecurity with a dichotomous measure (yes/no) of monthly ability to pay for rent/mortgage. We measured two types of stigma and discrimination: HIV-related and racial. We used the 10-item “Stigma Scale Revised” to measure multidimensional forms of HIV-related stigma, including personalized, disclosure concerns, negative self-image, and public attitudes. [90, 91] Cronbach’s αs were: personalized stigma subscale = 0.89, disclosure subscale = 0.76, negative self-image subscale = 0.87, public attitudes subscale = 0.76. We adapted the 8-item “Everyday Discrimination Scale” items from measuring general discrimination (‘In your day-to-day life how often have any of the following things happened to you?’) to assess racial discrimination (based on your race) (Cronbach’s α = 0.91). [92]

Social support was assessed using the 19-item MOS Social Support Survey that included emotional, informational, tangible, affectionate, and positive social interaction sub-scales. [93] Social support access was assessed on a 5-point scale. Cronbach’s αs were: emotional support subscale = 0.92, informational support subscale = 0.79, tangible support subscale = 0.86, affectionate support subscale = 0.61, positive social interaction subscale = 0.93. To assess depression we used the 7-item Beck Depression Inventory Fast-Screen (BDI-FS) to elicit a rapid assessment of affective and cognitive dimensions of depression (Cronbach’s α = 0.85). [94] We omitted the suicidality item due to lack of PRA training on mental health crises; prior studies found omission of this item did not alter scale reliability. [95] A single-item global self-rated health indicator recommended by the World Health Organization was used. [96]

### Statistical Analyses

We conducted descriptive statistics to calculate means and standard deviations of continuous variables, and frequencies of categorical variables. Scale items were summed in order to calculate total scale scores, and sub-scale scores where applicable. Scale reliability was assessed using Cronbach’s alpha. Non-missing responses were included in data analyses. There were 173 participants who completed the survey; we included 157 participants with complete data in the analyses. There were no significant differences in socio-demographics or outcome variables between participants included and excluded in the analyses. We conducted independent sample t-tests for continuous variables, and chi-square analyses for categorical variables, to examine differences across key outcome variables between persons who were, and were not, experiencing housing insecurity. We also assessed differences between African and Caribbean participants using independent sample t-tests and chi-square analyses.

Structural equation modeling (SEM) using maximum likelihood estimation was conducted to evaluate the hypothesized conceptual model (Fig 1). SEM can include observed as well as latent variables; we developed 2 latent variables (HIV-related stigma that included personalized, disclosure concerns, negative self-image, and public attitudes sub-scales; social support that included emotional, informational, tangible, affectionate and positive social interaction subscales). The observed variables of depression and racial discrimination were continuous variables, and housing insecurity categorical. Model fit was determined using: chi-square; Root-Mean Square Error of Approximation (RMSEA), where a score less than 0.08 indicates an acceptable fit; Comparative Fit Index (CFI), where a score greater than 0.90 is an acceptable fit, and the Tucker-Lewis Index (TLI) with a desired value of >0.90.[97] Analyses were conducted using IBM SPSS 22 and IBM AMOS 23.

## Results

We present descriptive statistics of participant characteristics in Table 1 by African and Caribbean ethnicity.

**Table 1:** Socio-demographic Characteristics of Survey Participants (n = 173) by Ethnicity in the African and Caribbean Black Women’s Stigma and Health Study: Ontario, Canada, 2010–2011.

Individual Variables		Mean (SD)*		
	**Overall sample mean score**	African participants (n = 73)*	Caribbean participants (n = 72)*	**p-value of differences**
Age (years) (n = 173)	40.7 (8.8)	40.2 (SD: 9.1)	40.9 (SD: 8.7)	t = 0.52 (df: 142), p = 0.61
Monthly income (US$)	3917.8 (11589.3); median: 1400.00 (range 0–7916)	3753.1 (SD: 12112.3)	4175.7 (SD: 11885.3)	t = 0.20 (df: 124), p = 0.84
		n (%)*	n (%)*	
		**African participants (n = 73)**	**Caribbean participants (n = 72)**	**p-value of differences**
Highest level of education completed				χ^2^(3, N = 137) = 2.98, p = 0.70
	Less than high school	16 (23%)	20 (29%)	
	High school	16 (23%)	20 (29%)	
	College diploma	23 (33%)	18 (27%)	
	University degree	14 (20%)	10 (15%)	
Location of residence				χ^2^ (5, N = 145) = 26.26, p<0.001
	Toronto	22 (30%)	50 (69%)	
	Ottawa	17 (23%)	8 (11%)	
	Windsor	8 (11%)	0 (0%)	
	Niagara Falls	8 (11%)	6 (8%)	
	Kitchener	9 (12%)	4 (6%)	
	Hamilton	9 (12%)	4 (6%)	
Relationship status				χ^2^ (6, N = 142) = 4.12, p = 0.66
	Single	27 (38%)	33 (49%)	
	Married	14 (20%)	12 (18%)	
	Separated	8 (11%)	11 (16%)	
	In a relationship/dating	11 (16%)	5 (7%)	
	Widowed	7 (10%)	5 (7%)	
	Common law/living with partner	2 (3%)	1 (1%)	
	Divorced	2 (3%)	1 (1%)	
Employment status				χ^2^ (4, N = 145) = 0.73, p = 0.95
	Part-time	9 (12%)	12 (17%)	
	Full-time	14 (19%)	11 (15%)	
	ODSP (disability)	37 (51%)	35 (49%)	
	Social assistance (welfare)	9 (12%)	10 (14%)	
	Canadian Pension Plan (retired)	4 (6%)	4 (6%)	
Income insecurity^+^				
	Rent/mortgage in full every month on time	14 (20%)	19 (28%)	χ^2^ (1, N = 140) = 1.19, p = 0.28
	Food each month	29 (40%)	36 (50%)	χ^2^ (1, N = 145) = 1.55, p = 0.21
	Medication costs not covered by other sources	21 (30%)	22 (33%)	χ^2^ (1, N = 136) = 0.18, p = 0.67
	Transportation costs every month	32 (44%)	31 (44%)	χ^2^ (1, N = 142) = 0.00, p = 0.99
	Childcare costs	34 (49%)	24 (36%)	χ^2^ (1, N = 136) = 2.52, p = 0.11
	Heating/cooling of your room/apartment/home	47 (64%)	46 (66%)	χ^2^ (1, N = 143) = 0.03, p = 0.87
	Supplements, or other forms of healthcare	47 (65%)	49 (70%)	χ^2^ (1, N = 142) = 0.36, p = 0.55
	Fun activities (i.e. movies, go out to dinner)	54 (74%)	46 (68%)	χ^2^ (1, N = 141) = 0.68, p = 0.41
Perceived poverty: “I think of myself as poor”				χ^2^(4, N = 141) = 8.23, p = 0.14
	Strongly disagree	3 (4%)	7 (10%)	
	Disagree	3 (4%)	9 (13%)	
	Neither agree nor disagree	17 (23%)	18 (27%)	
	Agree	29 (40%)	22 (32%)	
	Strongly agree	21 (29%)	12 (18%)	

^+^ Income insecurity assessed by participant reporting not enough income monthly to pay for each variable listed (i.e. rent, food).

*calculated from non-missing responses; participants were permitted to decline responding to items

In the overall sample, participants' (n = 173) mean age was 40.7 years (SD 8.8) and median monthly income was $1400.00 (range 0–7917). Out of 145 participants who reported their ethnicity, about half identified as African and half as Caribbean ethnicity; over two-thirds had completed high school or more; and two-thirds were receiving government assistance as their income, including disability, welfare or pension plans. Almost one-quarter of participants (n = 157) reported not having enough income to pay for rent/mortgage each month (22.5%; n = 39). There were no significant differences between African and Caribbean participants with the following variables: age, income, HIV-related stigma, social support, income security, including housing security, depression, or quality of life (not shown). African participants were more likely than Caribbean participants to live outside of Toronto, and to experience higher racial discrimination: t (138) = 3.14, p = 0.002, (CI: 1.48, 6.52) (not shown).

As presented in Table 2, participants reporting housing insecurity had significantly lower income and social support, and higher HIV-related stigma and depression scores, than participants with secure housing.

**Table 2:** Socio-demographic, Stigma, and Health Variables by Housing Insecurity Among Survey Participants in the African and Caribbean Black Women’s Stigma and Health Study: Ontario, Canada, 2011.

Variable		Experiencing Housing Insecurity (n = 157)			
		Yes (n = 39)	No (n = 118)	T-test/Chi-square test result	p value
Socio-demographics					
	Age (mean)	40.31	40.64	t(155) = 0.205, (CI: -2.89, 3.55)	0.84
	Income (mean)	1772.06	4729.84	t(155) = -2.52, (CI: -637.52, -5278.03)	0.01**
Ethnicity					
	African	14	57	χ^2^(1, N = 140) = 0.79	0.37
	Caribbean	19	50		
Education (n = 148)					
	High school or less	19	62	χ^2^(1, N = 148) = 0.23	0.63
	More than high school	18	49		
Place of Residence					
	Outside Toronto	18	56	χ^2^(1, N = 157) = 0.02	0.89
	In Toronto	21	62		
Stigma (mean)					
	HIV-related stigma	37.53	34.62	t(155) = 2.02, (CI: 0.07, 5.75)	0.04*
	Racial discrimination	31.01	28.93	t(155) = 1.41, (CI: -0.84, 5.00)	0.16
Health Outcomes (mean)					
	Depression	6.46	4.82	t(155) = 2.21, (CI: 0.17, 3.10)	0.03*
	Social support	50.92	59.60	t(155) = -2.64, (CI: -15.19, -2.17)	0.01**
	Self-rated health	3.08	3.37	t(155) = -1.49, (CI: -0.69, 0.09)	0.14

Total sample size = 173, but for these analyses, n = 157 for housing insecurity (unless otherwise specified for ethnicity and education) because of missing data on outcomes of interest; participants were permitted to decline responding to items.

*p<0.05;

**p≤0.01

**Fig 2:**
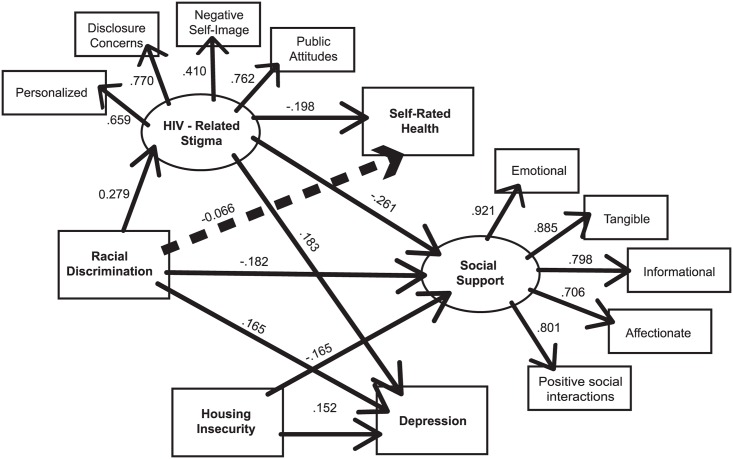
Final model of the relationship between HIV-related stigma, racial discrimination, housing insecurity and wellbeing among African and Caribbean women living with HIV in Ontario (n = 157). Fig 2 depicts the relationships between the latent variables (depicted as ovals) and observed variables (depicted as rectangles). Solid lines represent statistically significant direct effects and dotted lines represent statistically significant indirect effects. The standardized path coefficients next to each arrow reflect the strength and direction of the effect between variables, and the coefficient is similar to standardized beta weights in regression modeling.

The goal of the study was to develop a useful conceptual model of the relationships between HIV-related stigma, racial discrimination, housing insecurity, and wellbeing (depression, social support, self-rated health). The final model fit the data very well: χ^2^ (45, n = 154) = 54.28, p = 0.387; CFI = 0.997; TLI = 0.996; RMSEA = 0.016. The significant paths are presented in Fig 2. Paths in the final model were significant except for direct paths between: HIV-related stigma → housing insecurity; racial discrimination → housing insecurity; racial discrimination → SRH, and housing insecurity → SRH. Standardized and unstandardized coefficients are presented in Table 3.

**Table 3:** Parameter Estimates for Final Path Model in African and Caribbean Black Women’s Stigma and Health Study: Ontario, Canada, 2011 (n = 157).

Parameter	Coefficient (SE)	Critical Ratio	p	Standardized
Racial discrimination on				
*HIV-related stigma*	0.082 (0.025)	3.245	0.001	0.279
*Housing insecurity*	0.006 (0.005)	1.299	0.194	0.103
*Depression*	0.081 (0.035)	2.332	0.020	0.165
*Social support*	-0.024 (0.010)	-2.473	0.013	-0.182
*Self-rated health*	-0.070 (0.042)	-1.654	0.098	-0.132
HIV-related stigma on				
*Housing insecurity*	0.013 (0.018)	0.708	0.479	0.063
*Depression*	0.309 (0.146)	2.108	0.035	0.183
*Social support*	-0.116 (0.038)	-3.076	0.002	-0.261
*Self-rated health*	-0.357 (0.167)	-2.142	0.032	-0.198
Housing insecurity on				
*Depression*	1.275 (0.556)	2.251	0.024	0.152
*Social support*	-0.365 (0.155)	-2.353	0.019	-0.165
*Self-rated health*	0.012 (0.691)	0.018	0.986	0.001
Latent variables:				
Social support				
*Emotional support*	1.000			0.921
*Informational support*	0.906 (0.061)	14.857	<0.0001	0.885
*Tangible support*	0.869 (0.074)	11.686	<0.0001	0.798
*Affectionate support*	0.937 (0.084)	11.096	<0.0001	0.706
*Positive social interactions*	0.873 (0.064)	13.629	<0.0001	0.801
HIV-related stigma				
*Personalized stigma*	1.000			0.659
*Disclosure concerns*	0.685 (0.092)	7.463	<0.0001	0.770
*Negative self-image*	0.669 (0.146)	4.592	<0.0001	0.410
*Public attitudes*	0.671 (0.090)	7.443	<0.0001	0.762

SEM results are presented in Table 3. As hypothesized, racial discrimination had a significant direct effect on HIV-related stigma (standardized regression weight: 0.279, p = 0.005), depression (standardized regression weight: 0.164, p = 0.020) and social support (standardized regression weight: -0.182, p = 0.013). There was a significant indirect effect of racial discrimination on SRH via the mediator of HIV-related stigma (standardized regression weight = -0.066, p = 0.028).

HIV-related stigma had a significant direct effect on depression (standardized regression weight = 0.183, p = 0.035), social support (standardized regression weight = -0.261, p = 0.002), and SRH (standardized regression weight = -0.198, p = 0.032). Housing insecurity had significant direct effects on depression (standardized regression weight = 0.152, p = 0.024) and social support (standardized regression weight = -0.165, p = 0.019).

## Discussion

Current available literature assessing the impacts of housing insecurity on PLHIV focuses on risk practices, [65, 98] drug use, [65, 99] adherence to ARVs, [99, 100] and access to health care. [101] To our knowledge, only two qualitative studies have explored housing insecurity and its connection with stigma, discrimination and health outcomes among WLWH in Canada. [72, 74] Our study builds on these, using SEM techniques, to further conceptualize the relationships between stigma, housing insecurity and wellbeing of WLWH.

We found housing insecurity had direct effects on both increased depression and reduced social support outcomes. This finding is consistent with prior research reporting correlations between economic insecurity, including housing insecurity, and deleterious health outcomes, [59, 68, 69, 102, 103] such as higher rates of depressive symptoms. [68, 69, 104]. Research also highlights that poor health outcomes such as depressive symptoms are particularly high among WLWH [15, 105–107] who experience economic insecurity. [108] This finding is also congruent with fundamental cause theory that posits SES as a distal cause of disease. [86] ACB WLWH who experienced more housing insecurity reported lower social support, corroborating prior research that posits economic insecurity is a barrier to realizing social cohesion. [79] Poverty may be associated with smaller social networks and connections, and reduced positive interpersonal relationships. [86, 109] Social isolation, rejection and devaluation are fundamental components of stigma associated with poverty, HIV and other marginalized identity characteristics. [110] This extends previous work on poverty and social support to highlight the relationship between a particular form of poverty (housing insecurity) and social support among an understudied group, ACB WLWH. We did not find significant differences in self-rated health between participants who experienced housing insecurity and those who did not; this is an area for future research.

Experiencing racial discrimination was associated with a greater likelihood of experiencing HIV-related stigma. This highlights intersectional stigma, [9] the intersection of social exclusion based on multiple marginalized identity categories such as race and HIV positive serostatus. [9] Since the beginning of the epidemic social constructions of HIV as a ‘Haitian’ or ‘African’ disease reproduced racist stereotypes of Black persons rooted in notions of danger and sexual deviance. [111–115] Stigma may be higher for WLWH who are racialized. [116] Racism is embedded in HIV-related stigma as experienced by Black PLHIV, [117] suggesting the need for complex and multi-level interventions to tackle intersectional stigma. [9]

As hypothesized, racial discrimination and HIV-related stigma were associated with depression and lower social support, and HIV-related stigma was directly correlated with lower-self rated health and mediated the relationship between racial discrimination and self-rated health. The mediation finding suggests that women who experienced higher racial discrimination also experienced higher HIV-related stigma, and this increased HIV-related stigma directly contributed to poorer self-rated health. This reflects literature conducted among general populations that demonstrates harmful impacts of racism on health and wellbeing, [28] among PLHIV that illuminates the negative psychosocial and physical health effects of HIV-related stigma, [22, 27] particularly among racialized women who are already marginalized (e.g. based on race, sex and class). [11, 12, 106, 107]

Prior research indicates that culturally-related factors, such as strong racial or ethnic identity, [118] may buffer the impacts of racial and ethnic discrimination. [119, 120] Other research, including the minority stress model [121, 122], postulates that factors such as social support may reduce the influence of daily stressors from discrimination on wellbeing. The role of strong racial/ethnic identity as a protective factor may be influenced by other factors such as HIV-related stigma, and little is known about the relationship between social support and racial/ethnic identity among WLWH. Exploring both social support and strong racial/ethnic identity as potential mediators of this relationship among ACB WLWH warrants further investigation.

Contrary to our hypotheses, we did not find that racial discrimination or HIV-related stigma were significantly associated with housing insecurity. Rather, results suggest that ACB WLWH who were housing secure and housing insecure experienced similar levels of racial discrimination and HIV-related stigma. We employed measures of individual-level racial discrimination and HIV-related stigma, or of stigma and discrimination perceived by participants from other individuals. Future work may aim to include measures of structural racism and HIV-related stigma (e.g., federal policy, bank lending practices, real estate industry discrimination) to determine whether structural-level discrimination is associated with housing insecurity within this population. [31–33] Future research could also involve longitudinal analyses to better understand trajectories of housing insecurity and how these may be associated with changing levels of HIV-related stigma and/or racial discrimination following HIV diagnoses.

### Limitations

Our study design has limitations. First, our small non-probability sample limits generalizability of findings to other ACB WLWH. The purposive sampling design resulted in participants who had access to HIV services and organizational support, so we could have oversampled WLWH who were experiencing challenges with poverty and stigma. To produce generalizable findings with ACB WLWH, researchers should utilize random sampling to engage a representative sample of participants. Second, the cross-sectional nature of the study precludes assessment of causation, limiting our understandings of the exact nature of the relationship between economic insecurity, stigma and health. Causal pathways could be better clarified with a longitudinal study design. Third, we did not measure stigma associated with poverty, which could reproduce social exclusion. [110] Fourth, the racial discrimination scale focused on enacted stigma and did not assess the multi-dimensional aspects of stigma included in the HIV-related stigma scale. Conceptually stigma includes discriminatory processes but extends beyond this to integrate stereotypes and negative attitudes towards one’s groups. [123] Fifth, we aimed to recruit 200 participants but were not able to reach this target; this could be indicative of HIV-related stigma as a barrier to HIV research participation among ACB WLWH. If persons who experienced high levels of HIV-related stigma did not participate in this study, the study results may in fact underestimate the harmful effects of stigma on wellbeing among ACB WLWH. Finally, we aggregated data for African and Caribbean WLWH due to the small sample sizes in this study. African participants reported higher racial discrimination than Caribbean participants, and future research could further explore this phenomenon and its impact on health and wellbeing among WLWH.

### Conclusions

Our study has several strengths despite these limitations. To our knowledge this is the first study to examine associations between housing insecurity, racial discrimination, HIV-related stigma, and several indicators of wellbeing (social support, depression, self-rated health) among WLWH. Findings support the salience of housing insecurity as a fundamental cause of health, and highlight the utility of applying intersectionality theory [9] to better understand the unique experiences of HIV-related stigma, racial discrimination, and wellbeing among precariously housed ACB WLWH. The confluence of poverty, HIV-related stigma and racial discrimination appears to result in multiple pathways that contribute to poor wellbeing.

We highlight alarming rates of housing insecurity among ACB WLWH. This points to the urgent need for poverty reduction strategies and for understanding the root causes of economic insecurity. Approximately two-thirds of participants received government assistance, raising questions about social policies that may perpetuate institutionalized economic exclusion of PLHIV. Further attention to social policies and assessment of their impact on PLHIV’s wellbeing is warranted. [124]

In addition to addressing housing insecurity, preventive interventions could focus on reducing stigma and building social support. A recent systematic review on stigma reducing interventions for African and Black women highlighted limited interventions developed to reduce stigma among ACB WLWH, and none that addressed poverty, gender or racial discrimination. [23] While there were promising studies from the U.S. that demonstrated the potential to help ACB WLHW cope with stigma and promote self-care, the focus was on the individual. [23] Future interventions could address multiple forms of stigma—and how they are intertwined with poverty—at community and structural levels. Another recent systematic review reported that support group participation was correlated with reduced mortality and morbidity, improved retention in care, and higher QOL among PLHIV. [78] Future interventions could tailor social support groups for ACB WLHW, with a focus on reducing stigma, poverty and depression.

To conclude, our results underscore the salience of fundamental cause theory to explore experiences of housing insecurity among ACB WLWH. The significant pathway between racial discrimination and HIV-related stigma supports the examination of marginalization associated with multiple identities. Understanding the complex interactions between structural issues such as housing insecurity, community level stigma and social exclusion, and wellbeing among ACB WLWH can inform multi-level interventions to alleviate poverty, reduce stigma, and build social support networks.
